# The Nitrogen Content in the Fruiting Body and Mycelium of *Pleurotus Ostreatus* and Its Utilization as a Medium Component in Thraustochytrid Fermentation

**DOI:** 10.3390/bioengineering11030284

**Published:** 2024-03-16

**Authors:** Lina Schütte, Katharina Hausmann, Christoph Schwarz, Franziska Ersoy, Ralf G. Berger

**Affiliations:** 1 Institute of Food Chemistry, Gottfried Wilhelm Leibniz University Hannover, 30167 Hannover, Germanyfranziska.ersoy@lci.uni-hannover.de (F.E.);; 2 S2B GmbH & Co. KG, Zum Almsweg 2, 26197 Ahlhorn, Germany

**Keywords:** *Basidiomycota*, spent mycelium side stream, nitrogen source, medium substitution, thraustochytrids, triterpenes, squalene, bioprocess

## Abstract

Following the idea of a circular bioeconomy, the use of side streams as substitutes for cultivation media (components) in bioprocesses would mean an enormous economic and ecological advantage. Costly compounds in conventional media for the production of the triterpene squalene in thraustochytrids are the main carbon source and complex nitrogen sources. Among other side streams examined, extracts from the spent mycelium of the basidiomycete *Pleurotus ostreatus* were best-suited to acting as alternative nitrogen sources in cultivation media for thraustochytrids. The total nitrogen (3.76 ± 0.01 and 4.24 ± 0.04%, respectively) and protein (16.47 ± 0.06 and 18.57 ± 0.18%, respectively) contents of the fruiting body and mycelium were determined. The fungal cells were hydrolyzed and extracted to generate accessible nitrogen sources. Under preferred conditions, the extracts from the fruiting body and mycelium contained 73.63 ± 1.19 and 89.93 ± 7.54 mM of free amino groups, respectively. Cultivations of *Schizochytrium* sp. S31 on a medium using a mycelium extract as a complex nitrogen source showed decelerated growth but a similar squalene yield (123.79 ± 14.11 mg/L after 216 h) compared to a conventional medium (111.29 ± 19.96 mg/L, although improvable by additional complex nitrogen source).

## 1. Introduction

*P. ostreatus* is a widely cultivated edible mushroom from the division *Basidiomycota*. The fungus and its products are used in applications in the food, pharmaceutical and cosmetic industries [1]. Species from the *Pleurotus* genus contain high protein, mineral and vitamin contents as well as a low fat content. Their presumably health-protecting chemical composition and appealing flavor make them a valuable contribution to the human diet [1,2,3]. Due to their unique ability to secrete enzymes able to degrade lignocellulosic material, domestically grown *Basidiomycota* are cultivated on various side streams from the agriculture, woodland, farming and manufacturing industries [4]. Fruiting bodies of *P. ostreatus* specifically are produced on substrates like wheat straw, sawdust or leaves [5].

Submerged cultures of basidiomycetes, on the other hand, are mostly used for the industrial production of extracellular enzymes, exopolysaccharides and bioactive metabolites [6]. For example, *Basidiomycota* can be used for the production of laccases or colorants in stirred-tank bioreactors [7,8]. As the products are secreted and/or extracted, the remaining biomass produced during cultivation has no specific use and can be considered a side stream.

Side streams are by-products from different industry sectors which still contain valuable substances. Their utilization would be highly beneficial for moving industrial production processes closer to the goal of a circular economy. Due to its high carbohydrate, protein, mineral and vitamin contents, fungal mycelium has a high nutritional value. In particular, carbon and complex nitrogen sources are expensive components of conventional media for the cultivation of different microorganisms [9,10]. The usage of leftover mycelium as a nutrient source for the cultivation of other organisms would significantly reduce the respective cultivation costs and thus represent an enormous economic and ecological benefit for the biotechnology industry. The generation of yeast extract from brewer’s yeast, for instance, closely resembles this approach [11].

Complex solid side streams can only be utilized by organisms like *Basidiomycota*, which are capable of secreting enzymes to digest the substrate. For the cultivation of microorganisms in need of more accessible nutrients, side streams must be pretreated [12]. The most common methods used to release nutrients like carbon or nitrogen sources are heat or pressure treatments as well as hydrolyses (acidic, alkaline or enzymatic) and hot water extraction [13]. 

Thraustochytrids are unicellular marine eukaryotes which are of interest for the bio-technology industry due to their ability to accumulate high levels of lipids (up to 50% of their dry matter) [14]. Species of this family are potential sources for polyunsaturated fatty acids, terpenes, sterols and vitamins [15]. The triterpene squalene is a valuable product predominately used in the cosmetic and pharmaceutical industries. This polyunsaturated substance (C_30_H_50_) is mainly obtained from deep sea sharks and plants (e.g., olives, amaranth), which causes problems like overfishing and a high demand for arable land [16]. It was reported previously that thraustochytrids are able to grow on (preprocessed) side streams like food waste, forest biomass, glycerol, wastewater or cane molasses [12,16,17,18,19].

The aim of this work was to conduct a comparative analysis of the fruiting body and mycelium of *P. ostreatus* and their utilization as nitrogen sources for cultivation media for *Schizochytrium* sp. S31. For this, the total nitrogen and protein contents of the fungal components were determined. Afterward, different pretreatment methods were applied to freeze-dried fruiting bodies and the mycelium to generate a particle-free extract rich in complex nitrogen sources. As fruiting body remains can still be used in the food industry, only the mycelium extract from submerged cultivations of *P. ostreatus* was used in cultivations of the thraustochytrid *Schizochytrium* sp. S31 to evaluate its growth and squalene productivity. 

## 2. Materials and Methods

### 2.1. Reagents

All chemicals were purchased from Carl Roth (Karlsruhe, Germany), Sigma Aldrich (Darmstadt, Germany), Merck Millipore (Darmstadt, Germany), Riedel-de Haën AG (Seelze, Germany), Honeywell (Charlotte, NC, USA), VWR (Darmstadt, Germany) and Thermo Fisher Scientific (Waltham, MA, USA), unless stated otherwise. All organic solvents were bought from Carl Roth (Karlsruhe, Germany) and Supelco (Bellefront, PA, USA). All solvents were bought at analytical grade for gas chromatography (methanol and chloroform) or purified to analytical grade by in-house distillation (hexane).

### 2.2. Nitrogen and Protein Content Determination

The nitrogen content determination of freeze-dried fungal components was performed with a K-424 digestion unit and a K-350 distillation unit (BÜCHI Labortechnik AG, Flawil, Switzerland) according to the manual. The total nitrogen and protein contents were estimated following a modified Kjeldahl method [20]. Samples of freeze-dried *P. ostreatus* fruiting body or mycelium weighing 0.5 g were used for the analysis. The samples were hydrolyzed with concentrated sulfuric acid catalyzed by copper- and titan-containing tablets (Kjeldahl tablets, free of mercury and selenium, Carl Roth). The infusion of 8 M NaOH led to the release of ammonia, which was transferred to a flask containing 4% boric acid through steam distillation. Acidimetric back titration with 0.1 M HCl was performed to determine the total nitrogen present in the samples. Determinations were performed in triplicates. Considering the high non-proteinogenic nitrogen content in fungi, a conversion factor of 4.38 was used for the estimation of the total protein content [2].

### 2.3. Generation and Preparation of P. ostreatus Components

*Pleurotus ostreatus* fruiting bodies were bought at a local supermarket. The mycelium of the same species (strain *P. ostreatus* var. florida) was produced in-house. The fungus was grown and maintained on standard nutrient liquid agar (SNL, 30 g d-glucose monohydrate, 4.5 g l-asparagine monohydrate, 3 g yeast extract, 1.5 g KH_2_PO_4_, 0.5 g MgSO_4_, 5 µg Cu_S_O_4_ · 5 H_2_O, 80 µg FeCl_3_ · 6 H_2_O, 30 µg MnSO_4_ · H_2_O, 90 µg ZnSO_4_ · 7 H_2_O, 400 µg EDTA and 20 g agar per liter at a pH of 6.0) according to Sprecher [21]. The plates were incubated for 7 days at 24 °C before 1 cm^2^ of the overgrown agar was transferred into 150 mL of SNL medium (without agar) in a 300 mL Erlenmeyer shake flask as a preparatory culture. The culture was homogenized for 5 s at 11,000 min^−1^ (MiniBatch D-9, Miccra GmbH, Germany) and incubated on an orbital shaker with a shaking diameter of 25 mm for 7 days at 24 °C and 150 rpm. Then, 1.25% (*v*/*v*) of the preculture was used to inoculate the main culture (1 L of SNL (without agar) in 2 L shake flasks), which was incubated as described above. The biomass was harvested after 7 days and separated from the supernatant by centrifugation at 14,050 rcf (relative centrifugal force) for 10 min. The mycelium was washed thrice with demineralized water. The mycelium and fruiting bodies were freeze-dried (Alpha 1–4 LSCbasic, Martin Christ Gefriertrocknungsanlagen GmbH, Germany) and pulverized in a bead mill (MM400, Retsch GmbH, Germany) for 55 s at 30 Hz using a single 22 mm diameter steel bead. 

### 2.4. Fungal Component Extraction and Hydrolysis

The extraction/hydrolysis of the freeze-dried and pulverized fruiting body or mycelium was performed in a 100 mL shake flask containing 10 mL of water (sterile or non-sterile) with 5% (*w*/*v*) fungal component. For sterile conditions, the fungal component was dry-sterilized at 140 °C for 16 h before use. In case additional hydrolytic enzymes were added, 10 µL of Flavourzyme^®^ (1205 U/g, aminopeptidase units) and/or Viscozyme^®^ (108 U/g, ß-glucanase units) was added to the sample. The peptidase mix Flavourzyme^®^ was used for the protein/peptide hydrolysis of the fungal components, and the carbohydrase mix Viscozyme^®^ was used for the carbohydrate hydrolysis of the fungal components. All samples were incubated on an orbital shaker with a shaking diameter of 25 mm and a frequency of 150 rpm at 45 or 4 °C. Samples measuring 1 mL were taken after 0, 3, 6 and 24 h and centrifuged at 16,089 rcf for 10 min. Supernatants were inactivated by incubation on a thermal shaker (1.5 mm shaking diameter) at 95 °C and 300 rpm for 20 min. After filtration (cellulose–acetate, 0.45 µm), supernatants were stored at −4 °C until the analysis.

Acid hydrolysis (6 M HCl) of the freeze-dried and pulverized fungal components (5% w/v) was performed at 100 °C for 16 h. Afterward, the samples were neutralized with 6 M NaOH, filtered (cellulose–acetate, 0.45 µm) and stored at −4 °C until the analysis.

The mycelium extract for the cultivation of *Schizochytrium* sp. S31 was produced under non-sterile conditions in two 2 L shake flasks filled with 37.5 g of freeze-dried and pulverized mycelium and 750 mL of demineralized water each. The shake flasks were incubated at 45 °C and 150 rpm for 24 h. After incubation, the extract was separated from the side stream by centrifugation for 30 min at 14,050 rcf, followed by vacuum filtration (cellulose–acetate, 0.45 µm). The mycelium extract was autoclaved, and 1 mL samples were stored at −4 °C for d-glucose and free-amino-group quantification.

### 2.5. Cultivation of Schizochytrium sp. S31

*Schizochytrium* sp. S31 (American Type culture collection (ATCC) 20888) was preserved in 25% (*v*/*v*) glycerol with a preculture medium (PM, 10 g d-glucose, 1 g yeast extract, 1 g peptone ex casein, 12.5 g NaCl, 0.5 g KCl, 2.5 g MgSO_4_ · 7 H_2_O, 1 g monosodium glutamate, 6 mg ammonium iron (III) citrate, 51.5 mg CaCl_2_, 30.4 mg K_2_HPO_4_, 2.86 mg H_3_BO_3_, 1.81 mg MnCl_2_ · 4 H_2_O, 0.222 mg ZnSO_4_ · 7 H_2_O, 0.39 mg Na_2_MoO_4_ · 2 H_2_O, 0.079 mg CuSO_4_ · 2 H_2_O, 0.0477 mg CoSO_4_ · 7 H_2_O or 0.0494 mg Co(NO_3_)_2_ · 6 H_2_O, 0.1 mg carbenicillin, 595.8 mg HEPES, 0.5 µg biotin, 100 µg thiamine hydrochloride and 0.5 µg cobalamin per liter) at −80 °C.

50 mL of PM w/o agar was inoculated with cells from an agar plate (1.5% agar *w*/*v*) and incubated for two days at 28 °C. For precultures, 50 mL of PM w/o agar was inoculated with cells from an agar plate (1.5% agar *w*/*v*) and incubated for two days at 28 °C and 200 rpm with a shaking diameter of 25 mm. The media used for the main shake flask cultivations were the standard medium (M50: PM but with 60 g of d-glucose, 2.5 g of yeast extract and 2.5 g of peptone ex casein per liter), M50 without complex nitrogen sources (M50 − N: M50 without yeast extract and peptone ex casein) and M50 with additional complex nitrogen sources (M50 + N: M50 with 10.2 g of yeast extract and 10.2 g of peptone ex casein per liter), the mycelium extract without media supplements (E: mycelium extract with d-glucose added to a final concentration of 60 g, 0.1 mg of carbenicillin and 12.5 g of NaCl per liter) and M50 medium containing the mycelium extract as a complex nitrogen source (M50 − N + E: M50 without peptone ex casein and yeast extract but with mycelium extract). The pH values of the extract-containing media were adjusted to equal those of M50 (approximately 6.9) with NaOH. Then, 1 mL samples of the media were stored at −4 °C for free-amino-group quantification. An amount of 165 mL of each medium (500 mL shake flasks) was inoculated with 5% (*v*/*v*) of the preculture and incubated as described before. Samples measuring 1 (0 h) and 10 mL (24 h and after) were taken from the cultures every 24 h.

### 2.6. Determination of Dry Matter

Samples from the cultivation experiments were centrifuged at 4676 rcf for 10 min, and 1 mL of the supernatant was stored at −20 °C for d-glucose quantification. The pellet was washed once with an amount of demineralized water equal to the sample volume. Swimming cells were harvested and washed by vacuum filtration (cellulose–acetate, 0.45 µm). Cell pellets and/or filters were freeze-dried for at least 24 h (Alpha 1–4 LSCbasic, Martin Christ Gefriertrocknungsanlagen GmbH, Germany). Dry matter was determined gravimetrically.

### 2.7. Quantification of d-Glucose

The d-glucose in the fermentation supernatants and media was measured in duplicates using a d-glucose assay kit (GOPOD format, Megazyme, Ireland) according to the manufacturer’s protocol. External calibration with d-glucose standards (0–1 g/L) was performed.

### 2.8. Quantification of Free Amino Groups

The primary amino groups in the fermentation supernatants, media and extracts were detected in duplicates at 340 nm after derivatization with *ortho*-phthalaldehyde (OPA) according to the modified version [22] of the method established by Nielsen et al. [23]. External calibration with l-serine standards (0–5 mM) was performed.

### 2.9. Peptidase Activity Determination

The azocasein assay of Iversen and Jørgensen was slightly modified [24]. First, 100 µL substrate (5% azocasein in H_2_O), 375 µL buffer (0.1 M K_2_HPO_4_/KH_2_PO_4_; pH 6) and 25 µL sample were mixed and incubated for 20 min at 43 °C in a rotary shaker (1.5 mm shaking diameter) at 700 rpm. The reaction was stopped using 1 mL of trichloroacetic acid (3%). For the blanks, the enzyme sample was added after the trichloroacetic acid. Samples and blanks were subsequently stored on ice for 10 min and centrifuged at 15,000 rcf and 20 °C for 15 min. The absorbance of the supernatants was measured at 366 nm. One arbitrary unit (aU) is defined as the enzyme activity that increases the absorbance by 0.01 per min at 43 °C.

### 2.10. Squalene Extraction

Squalene extraction from the freeze-dried cells was performed in a two-step process. First, the total lipid content was extracted, followed by the purification of the unsaponifiable matter. The extraction of the total lipid content was performed as described by Bligh and Dyer [25]. Methanol, chloroform and 0.8% KCl were added to the freeze-dried cells in a ratio of 1:2:0.8 (*v*/*v*/*v*). After rigorous mixing for 30 min, chloroform and 0.8% KCl were added to obtain a final methanol–chloroform–0.8% KCl ratio of 2:2:1.8 (*v*/*v*/*v*). Phase separation was achieved by centrifugation (4676 rcf; 20 min). The lower phase was transferred to a new vial, and the chloroform was removed by evaporation under nitrogen. For the separation of unsaponifiable matter (containing squalene and sterols), 15% potassium hydroxide (*w*/*v*) in a methanol–water (4:1, *v*/*v*) mixture was added to the sample. Saponification of the samples was carried out at 60 °C for 3 h. Afterwards, the unsaponifiable matter was extracted three times with hexane, and the extract was stored at −20 °C until analysis. Phase separation was achieved each time by centrifugation (4676 rcf, 10 min). Octadecylbenzene (ODB) was used as an internal standard to monitor recovery.

### 2.11. Squalene Quantification

Squalene separation and quantification were achieved by gas chromatography. For GC measurements, a Shimadzu GC-2010 gas chromatograph equipped with a DB-5MS UI fused-silica capillary column (30 m × 0.32 mm; 0.25 µm film thickness), an AOC-20 s autosampler, a split/splitless injector and a flame ionization detector were used. Analyses were performed with a carrier gas (H_2_) flow of 5.18 mL/min and a split of 1:5. Injector and detector temperatures were set to 350 °C. Prior to the analysis, the samples were filtered through a 0.45 µm polytetrafluoroethylene (PTFE) membrane. The separation of 1 µL samples was performed by applying a temperature gradient starting at 130 °C (holding for 3 min) and increasing by 10 °C/min until 325 °C (holding for 10 min). Alkanes from C_21_ to C_40_ were used for the calculation of retention indices. Squalene quantification was performed by external calibration with analytical standards (1.24–123.50 mg/L). 

### 2.12. Statistical Analysis

OriginPro was used for the preparation of figures and statistical analyses. An unpaired two-sample Student’s *t*-test was used for the comparison of two groups (* *p* < 0.05; ** *p* < 0.01; *** *p* < 0.001), and a one-way ANOVA with a Tukey post hoc test (*p* < 0.05) was used for the comparison of more than two groups.

## 3. Results

### 3.1. Nitrogen and Protein Contents of P. ostreatus Fruiting Body and Mycelium

The total nitrogen and protein contents of the freeze-dried fruiting body and mycelium of *P. ostreatus* as well as the concentration of free amino groups after the acid hydrolysis of the fungal components are given in Table 1. The total nitrogen content determination was performed using the Kjeldahl method. The determination of free amino groups was performed by absorbance detection at 360 nm after OPA derivatization.

The total nitrogen and protein contents of the mycelium were 11% higher than those of the fruiting body. The determination of free amino groups after the acid hydrolysis of the freeze-dried fungus components resulted in a 10% higher value in the mycelium compared to the fruiting body. 

### 3.2. Hydrolysis and Extraction of Complex Nitrogen Sources from the Fruiting Body and Mycelium of P. ostreatus

**Figure 1:**
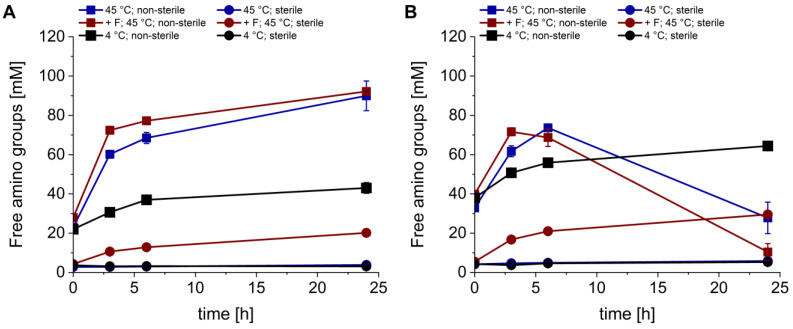
Hydrolysis of the mycelium (**A**) and fruiting body (**B**) of *P. ostreatus* and the aqueous extraction of the resulting free nitrogen sources. Experiments were conducted under non-sterile (squares) and sterile conditions (circles). Hydrolyses/extractions were performed at 45 °C (blue), at 45 °C with Flavourzyme^®^ (red, +F) and at 4 °C (black). Data presented are the means of triplicates ± standard deviations. Significant differences (*p* < 0.05) are denoted by lowercase letters depicted in Table S1 (one-way ANOVA; Tukey post hoc test).

The freeze-dried components of *P. ostreatus* were hydrolyzed and extracted in water under different conditions to obtain an aqueous extract rich in complex nitrogen sources. The concentration of free amino groups was monitored at different time points (0, 3, 6 and 24 h) over the course of the hydrolysis/extraction to monitor the release of complex nitrogen sources (Figure 1).

In general, the release of free amino groups was lower under sterile conditions compared to non-sterile conditions. In the sterile samples, a rise in the free amino group concentration was detected only after addition of Flavourzyme^®^. The hydrolysis and extraction of the mycelium was more successful at 45 than at 4 °C. Thus, the highest free amino group concentration was reached in the non-sterile samples after 24 h at 45 °C with and without addition of Flavourzyme^®^ (92.13 ± 1.26 and 89.93 ± 7.54 mM, respectively). 

During the first 6 h of the extraction, similar observations were made for the fruiting body. The free amino group concentration was maximal at 45 °C after 3 h with Flavourzyme^®^ and after 6 h without Flavourzyme^®^ (71.62 ± 2.02 and 73.63 ± 1.19 mM, respectively). After peaking, the free amino group concentrations in those samples dropped to 10.42 ± 4.26 mM (45 °C + Flavourzyme^®^) and 27.79 ± 8.02 mM (45 °C) after 24 h. The non-sterile samples incubated at 4 °C did not show a drop in the free amino group concentration and reached a maximum value of 64.39 ± 1.50 mM after 24 h.

**Figure 2:**
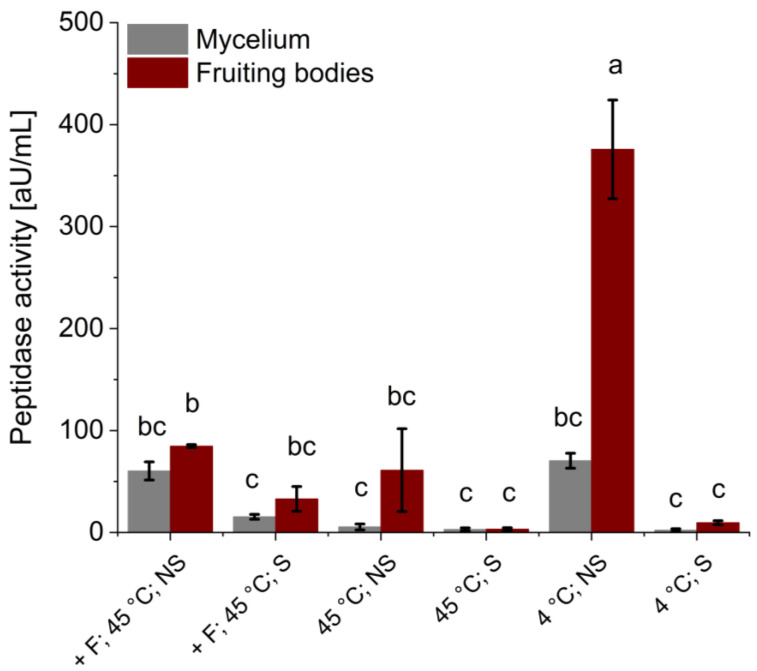
Remaining peptidase activity after the hydrolysis and extraction of *P. ostreatus* mycelium (grey) and fruiting body (red). Experiments were conducted under non-sterile (NS) and sterile conditions (S). Hydrolyses/extractions were performed at 45 °C, at 45 °C with Flavourzyme^®^ (+ F) and at 4 °C. Data presented are the means of triplicates ± standard deviations. Significant differences (*p* < 0.05) are denoted by lowercase letters (one-way ANOVA; Tukey post hoc test).

After hydrolysis and extraction, the remaining endo-peptidase activity was determined using azocasein as a substrate (Figure 2). In the sterile samples, no or very low (with Flavourzyme^®^) peptidase activity was detected. In the non-sterile samples, the highest peptidase activities of the mycelium samples were determined at 45 °C (with Flavourzyme^®^) and 4 °C (60.33 ± 8.92 and 70.41 ± 7.31 aU/mL, respectively). Non-sterile fruiting body samples hydrolyzed and extracted at 45 °C (with and without Flavourzyme^®^) had similar remaining peptidase activities of 84.74 ± 1.50 and 61.13 ± 40.68 aU/mL, respectively. The highest peptidase activity overall was found in the non-sterile fruiting body sample incubated at 4 °C (375.78 ± 48.47 aU/mL).

### 3.3. Utilization of P. ostreatus Mycelium Extract as a Complex Nitrogen Source

Incomplete or destroyed fruiting bodies of *P. ostreatus* can still be used in the food industry and can only be considered a side stream as part of left-over food. The fungal mycelium remaining after submerged cultivation, on the other hand, is often not produced under food-grade conditions. Additionally, in many countries, the consumption of the mycelium requires pre-market authorization (European Union) [26]. Therefore, only the mycelium of *P. ostreatus* can be considered a true side stream.

For the utilization of the fungal mycelium as a nitrogen source, it was thus hydrolyzed and extracted using the optimal method (non-sterile, 45 °C, without Flavourzyme^®^ and 24 h; cf. Section 3.2). The extract was to be used as a replacement for the complex nitrogen source in a conventional medium for the cultivation of a thraustochytrid (*Schizochytrium* sp. S31). For a comparative cultivation of this strain, different media containing 6% glucose were prepared: a conventional cultivation medium (M50: 0.25% peptone and 0.25% yeast extract), M50 medium without complex nitrogen sources (M50 − N), M50 medium with mycelium extract replacing peptone and yeast extract (M50 − N + E) and a mycelium extract with additional glucose, NaCl and carbenicillin (E). 

**Figure 3:**
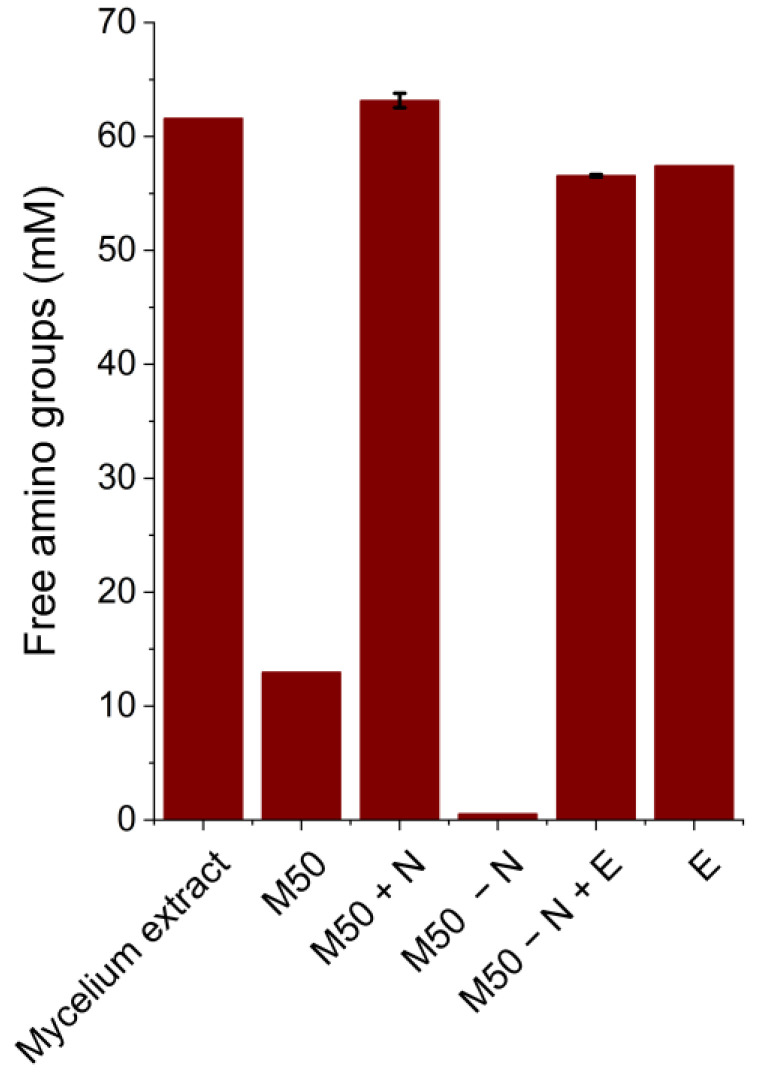
Free amino group concentration in media prepared for thraustochytrid cultivation. For media preparation, the chosen extraction/hydrolysis conditions of the mycelium (no Flavourzyme^®^, 45 °C and non-sterile) were upscaled. The media analyzed were a pure mycelium extract, a conventional medium (M50), M50 with an additional complex nitrogen source (M50 + N), M50 without a complex nitrogen source (M50 − N), M50 with complex nitrogen sources replaced by the mycelium extract (M50 − N + E) and the mycelium extract with added glucose, NaCl and carbenicillin (E). Data presented are the means of duplicates ± standard deviations.

To ensure equal amounts of nitrogen sources were present in the different media, free amino groups were determined. Their concentrations in the mycelium extract and the media containing the extract (M50 − N + E; E) were close to 60 mM (Figure 3). The lower free-amino-group yield in the mycelium extract compared to the hydrolysis and extraction experiments depicted in Figure 2 (under the same conditions: no Flavourzyme^®^, 45 °C and non-sterile) could be due to the upscaling of the experiment. Reduced mass transfer and mixing due to the larger liquid volume might have affected the hydrolysis efficiency negatively. In comparison to media containing the mycelium extract, fewer amino groups (12.98 mM) can be found in the conventional medium (M50). Therefore, the conventional medium, which equaled the amount of free amino groups present in the mycelium extract and the corresponding media, was prepared as a control (M50 + N: 1.02% peptone and 1.02% yeast extract). As expected, the M50 medium without the complex nitrogen source contained very few detectable free amino groups.

### 3.4. Cultivation of Schizochytrium sp. S31 on Mycelium Extract

The media described above were used for a comparative cultivation of the thraustochytrid strain *Schizochytrium* sp. S31 with the aim of high growth and squalene productivity. 

**Figure 4:**
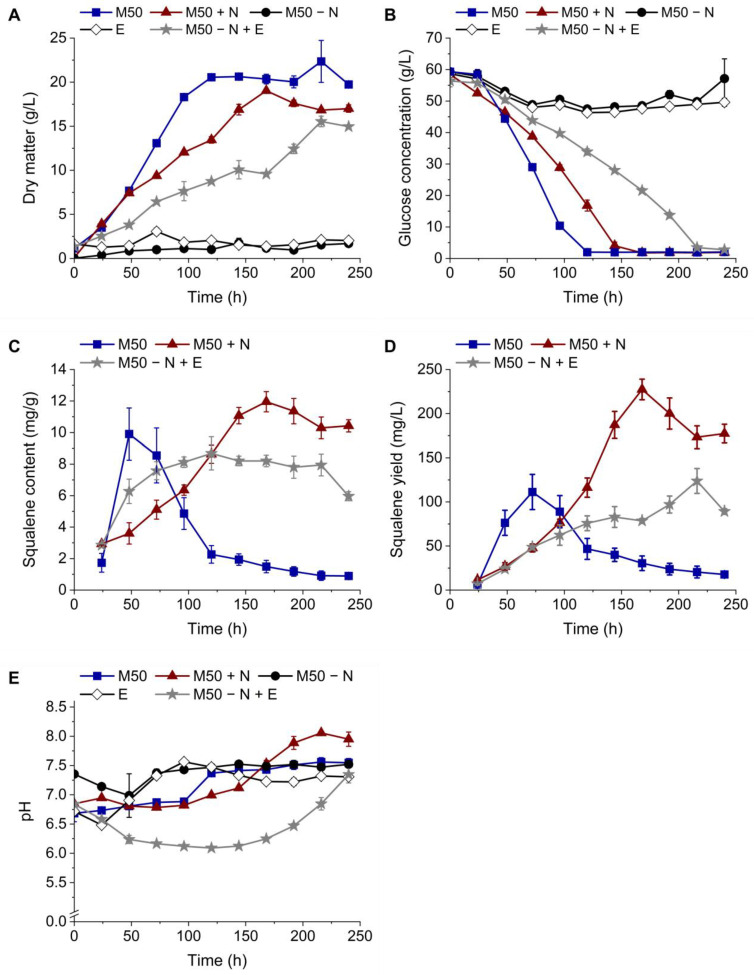
Effect of the substitution of complex nitrogen sources with a mycelium extract on the biomass formation and squalene accumulation in *Schizochytrium* sp. S31. The M50 medium is depicted in blue, the M50 medium with additional complex nitrogen source is depicted in red (M50 + N), the M50 medium without a complex nitrogen source is depicted in black (M50 − N), the mycelium extract with added glucose, NaCl and carbenicillin is depicted in white (E) and the M50 medium with the mycelium extract substituting the complex nitrogen source is depicted in grey (M50 − N + E). (**A**) Dry matter, (**B**) glucose concentration, (**C**) squalene content per gram of dry matter, (**D**) squalene yield per liter of cultivation volume and (**E**) pH over cultivation time. Data presented are the means of triplicates ± standard deviations. Significant differences (*p* < 0.05) were denoted by lowercase letters depicted in Table S2 (one-way ANOVA; Tukey post hoc test).

Figure 4A demonstrates that *Schizochytrium* sp. S31 did not grow on the M50 medium without complex nitrogen sources (M50 − N) or the mycelium extract (E), which was confirmed by the non-decreasing glucose concentrations over the course of the cultivation (Figure 4B). Thus, not enough biomass for squalene extraction and analysis was generated during those cultivations.

The conventional M50 medium led to the fastest growth and highest biomass, followed by the M50 medium with an additional nitrogen source (M50 + N) and the M50-medium with the mycelium extract as a complex nitrogen source replacement (M50 − N + E) (22.35 ± 2.38, 19.03 ± 0.12, 15.54 ± 0.60 g/L, respectively). Glucose concentrations decreased accordingly (Figure 4B). 

The squalene content (Figure 4C) of the cultivation in M50 peaked after 48 h (9.90 ± 1.65 mg/g) and decreased afterward over the course of three days. Cultivation in M50 + N and M50 − N + E led to delayed maximal squalene contents (11.95 ± 0.64 mg/g after 168 h and 8.68 ± 1.05 mg/g after 120 h, respectively). In contrast to the rapidly declining contents in M50, these remained relatively stable for the remainder of the cultivation. Squalene yield per liter of culture volume (Figure 4D) is influenced by biomass formation. Therefore, the highest yield was reached with the M50 + N medium after 168 h (227.40 ± 11.67 mg/L). The maximum yields of the cultivations in M50 and M50 − N +E were comparable (111.29 ± 19.96 and 123.79 ± 14.11 mg/L, respectively) but reached at different time points (after 72 and 216 h, respectively). 

The pH values (Figure 4E) oscillated between 6.5 and 7.5 except for the cultivations in M50 + N and M50 − N + E. In the stationary phase of the cultivation in M50 + N, the pH rose up to 8, and in M50 − N + E, it decreased to around 6 during the first 96 h and kept rising to a value of 7.3 at the end of the fermentation.

## 4. Discussion

An accurate comparison of the chemical composition of *Basidiomycota* is challenging due to its high variability depending on the growth substrate, strain, developmental state and microbial community [3]. As demonstrated in this work, the total nitrogen contents (dry weight) of the fruiting body (3.76 ± 0.01%) and mycelium (4.24 ± 0.04%) of *P. ostreatus* were highly similar. Hadar and Cohen-Arazi [27] reported similar values for *P. ostreatus* ‘Florida’; however, their nitrogen content of the fruiting body (4.0 + 0.05%) was higher than that of the mycelium (3.8 ± 0.13%). 

The protein contents (dry weight) of *P. ostreatus* reported in other sources range from 11 to 36% (fruiting body) [27,28,29,30,31,32] and from 21 to 40% (mycelium) [25,26,29]. It must be considered that the protein contents of mushrooms reported in the literature vary significantly due to the usage of different analytical methods or conversion factors in the case of the Kjehldahl analysis [33,34]. Additionally, the protein content of the fruiting body depends on the analyzed part and its growth phase. For instance, the stalk usually contains more proteins than the cap [32]. The protein content of the fruiting body determined here (16.47 ± 0.06%) fitted well into the range described in the literature, whereas that of the mycelium was slightly lower (18.57 ± 0.18%).

Solid side streams used for the cultivation of microorganisms interfere with dry matter determination and the extraction of intracellular products. To circumvent these challenges, aqueous extracts of the solid fungal components were generated. Similar to the generation of yeast extract from brewer’s or baker’s yeast [11], the hydrolysis and extraction were supposed to be cost- and energy-efficient. Even though *P. ostreatus* dry matter consists of approximately 40–70% carbohydrates [33,35], the release of free glucose and other reducing sugars from the fruiting body was low compared to the amount of carbon source used in conventional cultivation media (Figure S1). For these tests, the enzyme mix Viscozyme^®^ was chosen because it was found to be the most effective compared to other carbohydrase mixes in preliminary tests.

Therefore, this work focused on the substitution of expensive complex nitrogen sources like yeast extract and peptone using potential side streams from *Basidiomycota*. The release of free amino groups was found to be higher in non-sterile samples compared to their sterile equivalents (Figure 1). Sterile samples without Flavourzyme^®^ even showed no detectable rise in free amino group concentrations over the course of the experiment. These findings suggest that the reason for the release of free amino groups is not only the extraction process but also the hydrolysis of the freeze-dried fungal components. Pulverized lyophilizates of the fruiting body and mycelium apparently contained active peptidases which released free amino groups if they were not denatured by the sterilization process beforehand. Different sources described that basidiomycetes produce intra- and extracellular peptidases responsible for the regulation of enzymatic activity or nutrient acquisition [36,37,38]. Furthermore, it was reported by Krahe et al. [39] that enzymes present in a fungal mycelium can endure the freeze-drying process whilst remaining active, which is supported by the findings in this work. Decreasing free amino group concentrations in the non-sterile fruiting body samples incubated at 45 °C were likely caused by their consumption due to microbial contamination. Compared to the acid hydrolysis depicted in Table 1, 85 (mycelium) and 75% (fruiting body) of the maximal free amino group concentration was released through enzymatic hydrolysis, which demonstrates the efficiency of this cost- and energy-efficient process. 

The peptidase activity assay performed after the extraction/hydrolysis (Figure 2) confirmed that little to no activity was left in the sterile samples. The peptidase activities of the non-sterile samples, on the other hand, did not align with the concentration of free amino groups as the maximum activity was found in the fruiting body experiments performed at 4 °C. The reason for the low peptidase activity in the other non-sterile samples is most likely the denaturation of the enzymes over time due to the relatively high temperature of 45 °C.

Although only the mycelium can be considered a true side stream, the generation of extracts from the fruiting body is still relevant. The demonstration of the autolytic properties of the lyophilized fruiting body contributes to the understanding of fungal enzymes secreted for nutrient digestion. Additionally, the fruiting bodies of edible mushrooms may become relevant as side streams as part of left-over foods.

The utilization of extracts from other side streams like macro- and microalgae biomass were not successful. The generation of extracts from *Saccharina latissima* was challenging due to its high viscosity, and extracts from *Chlorella sorokiniana* inhibited the growth of the thraustochytrid (Figure S3) and thus, were not tested as a complex nitrogen source substitute. 

Similar to yeast extract from brewer’s or baker’s yeast, mycelium extracts from the submerged cultivation of higher fungi might be excellent sources of nitrogen and other nutrients. The utilization of a fungal mycelium from a previous bioprocess as a nutrient replacement is an important step in moving the biotechnology industry toward a circular economy. In this work, its use as a nitrogen source in the cultivation of thraustochytrids served as an exemplary application. 

Thus, for thraustochytrid cultivation, mycelium extraction and hydrolysis conditions yielding a high free amino group concentration (45 °C; non-sterile; 24 h) were chosen. The extract was used as a medium on its own but also to substitute the complex nitrogen source in a conventional medium. The results depicted in Figure 3 show that the mycelium extract and the resulting media contain an over 4-fold higher nitrogen source concentration compared to the M50 medium. The C/N ratio of the cultivation medium heavily influences lipid accumulation in thraustochytrids [40,41,42]. Therefore, cultivations on an M50 medium without a complex nitrogen source and on M50 with additional peptone and yeast extract to equal the free amino group concentration of the medium containing the mycelium extract were performed as a control. 

A comparative cultivation of the thraustochytrid *Schizochytrium* sp. S31 was executed in shake flasks (Figure 4). For cultivations in M50 − N and in E, no significant growth was observed, which was confirmed by non-decreasing glucose levels. As expected, the thraustochytrid did not grow without complex nitrogen sources. Stunted growth on the E medium is most likely explained by missing nutrients other than the carbon or nitrogen sources like sulfur, phosphate or salts other than NaCl [43]. Growth on M50 + N was slower compared to M50, which was likely caused by the excess nitrogen source. Zhang, et al. [44], for instance, reported that concentrations over 20 g/L effected the growth of *Aurantiochytrium*. sp. TWZ-97 negatively. Cultivations on M50 with the mycelium extract as a replacement for yeast extract and peptone led to even slower growth and less biomass production compared to M50 + N. In addition to nitrogen source excess and inhibiting substances from the mycelium, the significantly lower pH could have been the cause. Future work using N-rich side stream extracts could test the dilution of the extract to reach a concentration of free amino groups that equals that in the original medium in contrast to the approach used to increase the nitrogen content that was evaluated here.

Because of the acidic nature of the mycelium extract, the pH of the media containing the mycelium extract was adjusted to equal that of M50 before inoculation (Figure 4E). As presented in Figure S2, *Schizochytrium* sp. S31 showed a long lag-phase and weak growth on media containing mycelium extract without an adjusted pH. Although the pH was initially adjusted, it dropped to approximately six during the cultivation in M50 − N + E, which might have caused slower growth and stunted biomass formation [45,46]. Continuous pH adjustment throughout the course of cultivation might alleviate this problem and improve growth as well as squalene production further.

The squalene content in the thraustochytrid cells depended heavily on the amount of complex nitrogen source in the medium. While it peaked and dropped early in cultures grown on the M50 medium, the content stayed more stable on M50 + N and M50 − N +E. This can be explained by the higher concentrations of complex nitrogen sources in these media. Squalene accumulation seems to benefit from high contents of certain complex nitrogen sources, especially yeast extract [44,47]. As complex nitrogen sources also contain low amounts of sugars, fats, inorganic ions, vitamins and growth factors, it is difficult to identify the substance(s) directly responsible for the boosted squalene accumulation [47]. Reasons for the reduced squalene content in M50 − N + E compared to M50 + N could be similar to those responsible for the reduced growth (inhibitors, pH and missing nutrients). As the squalene yield per liter of culture volume is biomass-dependent, the highest value was found in the M50 + N cultivations (227.40 ± 11.67 mg/L), which was approximately twice as high as in the M50 and M50 − N + E cultivations.

The utilization of the mycelium as a nitrogen source will be even more beneficial after further optimization. For example, the optimal concentration of the mycelium extract has to be determined to control complex nitrogen source entry into the medium. This could prevent stunted growth due to an initial excess of a complex nitrogen source. Additionally, the pH must be managed precisely to prevent negative consequences of the addition of the acidic mycelium extract on the performance of the production organism (pH control; the addition of more or a different buffer to the medium). 

To the best of our knowledge, the only side streams used specifically for nitrogen source replacement reported in the literature were tofu whey wastewater or sorghum distillery residue for DHA and astaxanthin production in thraustochytrids, respectively [18,48]. As different strains, overall cultivation conditions and target molecules were observed in these studies, a usability comparison with the mycelium extract utilized in this work is challenging. Patel et al. [17] used food waste hydrolysates preliminary as a carbon source replace-ment for the co-production of DHA and squalene. However, they assumed an addi-tional positive effect of the nitrogen content (not determined) in the food waste on growth and squalene production (max. 1.05 g/L) of *Aurantiochytrium* sp. T66.

In conclusion, after further optimization, potential side streams from the cultivation of *Basidiomycota* may be utilized for the substitution of cultivation media components for other bioprocesses. The reutilization of nutrients plays an important role in the formation of a circular economy. Freeze-dried fungal components contain a high protein content and active peptidases, which enable the release of accessible nitrogen sources to an aqueous extract. Thus, extracts, especially from a fungal mycelium produced via submerged cultivation, might be a cost-efficient alternative for commonly used complex nitrogen sources (e.g., yeast extract and peptone).

## Figures and Tables

**Table 1:** Total nitrogen and protein contents and free amino groups after the acid hydrolysis of the freeze-dried fruiting body and mycelium from *P. ostreatus*. Acidic hydrolysis was performed as a reference for evaluating the hydrolysis efficiency of enzymatic hydrolyses. Data presented are the means of triplicates ± standard deviations. Significant differences are denoted by significance stars (unpaired two-sample Student’s *t*-test).

Sample	Total Nitrogen (%)	Total Protein (%) (N × 4.38)	Free Amino Groups (mM) after Acid Hydrolysis
Fruiting body	3.76 ± 0.01	16.47 ± 0.06	96.56 ± 1.63
Mycelium	4.24 ± 0.04 ***	18.57 ± 0.18 ***	107.84 ± 0.44 ***

*** *p* < 0.001.

## Data Availability

The datasets generated during and/or analyzed during the current study are available from the corresponding author on reasonable request.

## Supplementary Materials

The following supporting information can be downloaded at https://www.mdpi.com/article/10.3390/bioengineering11030284/s1, Figure S1: Hydrolysis of fruiting body of *P. ostreatus* and aqueous extraction of resulting free sugars.; Figure S2: Effect of substitution of complex nitrogen sources by mycelium extract (non-adjusted pH) on biomass formation and squalene accumulation in *Schizochytrium* sp. S31.; Figure S3: Effect of a *Chlorella sorokiniana* extract on biomass formation of *Schizochytrium* sp. S31.; Table S1: Hydrolysis of mycelium and fruiting body of *P. ostreatus* and aqueous extraction of resulting free nitrogen sources.; Table S2: Effect of substitution of complex nitrogen sources by mycelium extract on biomass formation and squalene accumulation in *Schizochytrium* sp. S31.
