# Supplementary material for: The Nitrogen Content in the Fruiting Body and Mycelium of Pleurotus Ostreatus and Its Utilization as a Medium Component in Thraustochytrid Fermentation
Source: Bioengineering (Basel). 2024 Mar 16;11(3):284. doi: 10.3390/bioengineering11030284 (PMC10968387; doi:10.3390/bioengineering11030284)
Supplement: Supplementary file 1 [file bioengineering-11-00284-s001.zip › bioengineering-2896986-supplementary.pdf]

Supplementary Materials

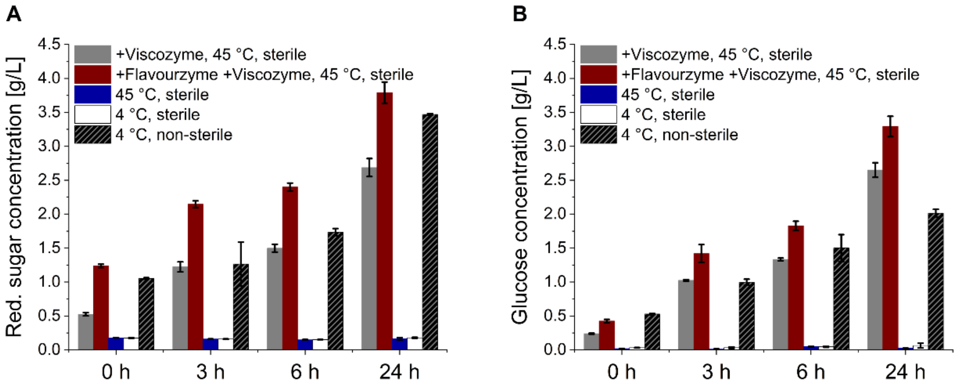

Figure S1. Hydrolysis of fruiting body of *P. ostreatus* and aqueous extraction of resulting free sugars. Hydrolyses/extractions were performed at 45 °C under sterile conditions (blue), at 45 °C with Viscozyme® (grey) and Flavourzyme® (red) and at 4 °C under non-sterile (black) and sterile conditions (white). (A) Reducing sugar concentration and (B) glucose concentration over time. Data presented are the means of triplicates ± standard deviations.

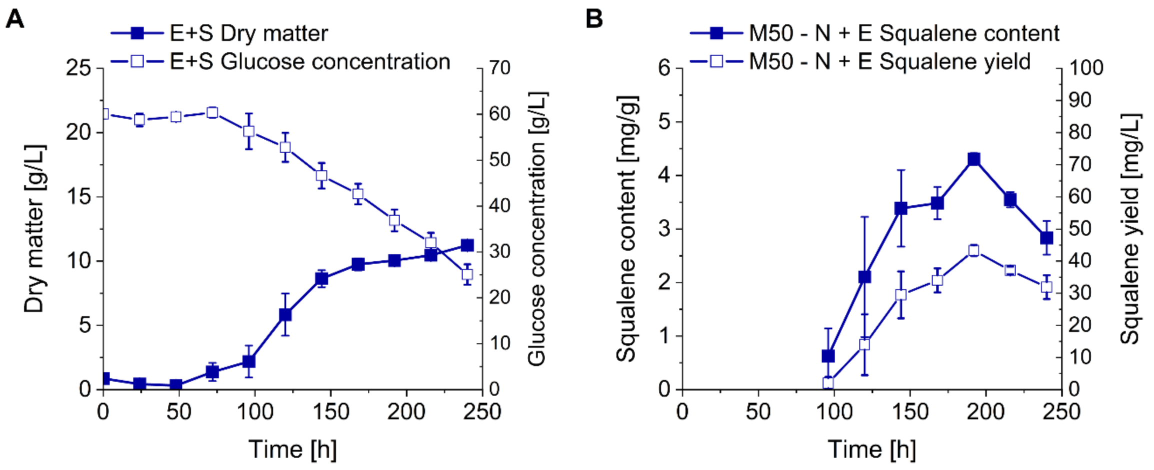

Figure S2. Effect of substitution of complex nitrogen sources by mycelium extract (non-adjusted pH) on biomass formation and squalene accumulation in *Schizochytrium* sp. S31. (A) Dry matter (blue) and glucose concentration (white) over time. (B) Squalene content per gram dry matter (blue) and squalene yield per liter cultivation volume (white) over time. Data presented are the means of triplicates ± standard deviations.

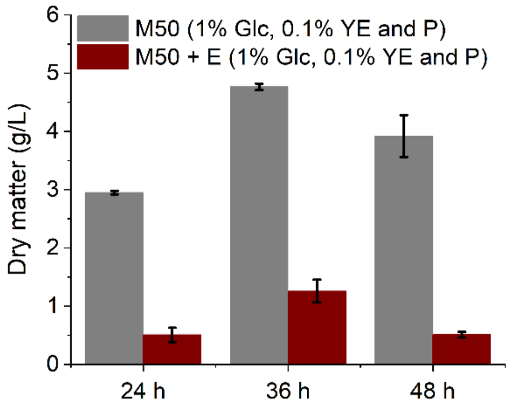

Figure S3. Effect of a *Chlorella sorokiniana* extract on biomass formation of *Schizochytrium* sp. S31. The extract from *Chlorella sorokiniana* was generated according to those of the fungal components at 4 °C for 16 h without hydrolytic enzymes. The extract was added to the M50 medium (0.1% yeast extract (YE) and peptone ex casein (P)) with a reduced D-glucose (Glc) concentration to achieve a combined D-glucose concentration of 1% (M50 + E). M50 medium (1% D-glucose, 0.1% yeast extract and peptone ex casein) was used as a control. Data presented are the means of duplicates ± standard deviations.

Table S1. Hydrolysis of mycelium and fruiting body of *P. ostreatus* and aqueous extraction of resulting free nitrogen sources. Experiments were conducted at 4 and 45 °C with and without Flavourzyme (+F) under sterile (S) and non-sterile (NS) conditions. Data presented are the means of triplicates ± standard deviations. Significant differences ($p < 0.05$) are denoted by lowercase letters in case of significantly different means (one-way ANOVA; Tukey post hoc test).

Sample	Free amino groups (mM)			
	0 h	3 h	6 h	24 h
**Fruiting body**				
+ F; 45 °C; NS	39.79 ± 1.03 [c]	71.63 ± 2.02 [a]	68.71 ± 4.58 [a]	10.42 ± 4.26 [b]
+ F; 45 °C; S	5.47 ± 1.24 [d]	16.73 ± 1.09 [c]	20.97 ± 0.93 [b]	29.49 ± 0.84 [a]
45 °C; NS	32.85 ± 1.08 [b]	61.70 ± 2.76 [a]	73.63 ± 1.19196[a]	27.79 ± 8.024[b]
45 °C; S	4.17 ± 0.35	4.66 ± 0.53	4.90 ± 0.56	5.82 ± 0.51
4 °C; NS	37.95 ± 0.97 [d]	50.73 ± 0.56 [c]	55.90 ± 0.57 [b]	64.39 ± 1.50 [a]
4 °C; S	4.29 ± 0.13	3.73 ± 1.17	4.64 ± 0.18	5.30 ± 0.39
**Mycelium**				
+ F; 45 °C; NS	27.93 ± 0.73 [d]	72.41 ± 1.60 [c]	77.23 ± 1.96 [b]	92.13 ± 1.26 [a]
+ F; 45 °C; S	4.28 ± 0.15 [d]	10.62 ± 0.12 [c]	12.82 ± 0.25 [b]	20.14 ± 0.19 [a]
45 °C; NS	23.00 ± 0.10 [c]	60.20 ± 0.05 [b]	68.49 ± 2.82 [b]	89.93 ± 7.54 [a]
45 °C; S	2.79 ± 0.15 [b]	2.83 ± 0.25 [b]	3.00 ± 0.25 [b]	3.82 ± 0.24 [a]
4 °C; NS	22.01 ± 0.48 [d]	30.62 ± 1.08 [c]	36.95 ± 1.40 [b]	43.00 ± 2.71 [a]
4 °C; S	3.66 ± 0.52	3.13 ± 0.19	3.22 ± 0.25	3.10 ± 0.31

Table S2. Effect of substitution of complex nitrogen sources by mycelium extract on biomass formation and squalene accumulation in *Schizochytrium* sp. S31. Data presented are the means of triplicates ± standard deviations. Significant differences ($p < 0.05$) are denoted by lowercase letters depicted in Table S2 (one-way ANOVA; Tukey post hoc test).

	**M50**	**M50 + N**	**M50 - N**	**M50 – N +E**	**E**
**Dry Matter (g/L)**					
0 h	1.13 ± 0.41 ^{e}	0.08 ± 0.01 ^{g}	0.02 ± 0.35 ^{c}	1.33 ± 0.37 ^{g}	1.63 ± 0.26 ^{bcd}
24 h	3.49 ± 0.12 ^{e}	3.91 ± 0.07 ^{g}	0.38 ± 0.09 ^{bc}	2.53 ± 0.10 ^{fg}	1.27 ± 0.05 ^{d}
48 h	7.68 ± 0.14 ^{d}	7.41 ± 0.14 ^{f}	0.86 ± 0.25 ^{abc}	3.80 ± 0.10 ^{f}	1.43 ± 0.02 ^{bcd}
72 h	13.08 ± 0.31 ^{c}	9.37 ± 0.25 ^{e}	0.99 ± 0.15 ^{ab}	6.43 ± 0.20 ^{e}	3.06 ± 0.39 ^{a}
96 h	18.31 ± 0.06 ^{b}	12.04 ± 0.06 ^{d}	1.12 ± 0.23 ^{ab}	7.63 ± 1.09 ^{de}	1.82 ± 0.07 ^{bcd}
120 h	20.55 ± 0.21 ^{ab}	13.45 ± 0.37 ^{c}	1.02 ± 0.16 ^{ab}	8.74 ± 0.17 ^{cd}	2.05 ± 0.19 ^{bc}
144 h	20.63 ± 0.03 ^{ab}	16.88 ± 0.61 ^{b}	1.74 ± 0.54 ^{a}	10.06 ± 1.05 ^{c}	1.53 ± 0.15 ^{bcd}
168 h	20.35 ± 0.54 ^{ab}	19.03 ± 0.12 ^{a}	1.16 ± 0.16 ^{ab}	9.57 ± 0.11 ^{c}	1.37 ± 0.06 ^{cd}
192 h	20.03 ± 0.68 ^{ab}	17.60 ± 0.37 ^{b}	0.96 ± 0.07 ^{ab}	12.43 ± 0.54 ^{b}	1.55 ± 0.14 ^{bcd}
216 h	22.35 ± 2.38 ^{a}	16.83 ± 0.22 ^{b}	1.54 ± 0.22 ^{a}	15.54 ± 0.60 ^{a}	2.11 ± 0.13 ^{b}
240 h	19.74 ± 0.14 ^{ab}	17.00 ± 0.42 ^{b}	1.69 ± 0.12 ^{a}	14.95 ± 0.20 ^{a}	2.03 ± 0.33 ^{bc}
**Glucose concentration (g/L)**					
0 h	59.31 ± 0.50 ^{a}	58.47 ± 0.21 ^{a}	59.30 ± 1.11 ^{a}	56.39 ± 1.88 ^{a}	58.68 ± 0.94 ^{a}
24 h	58.42 ± 1.60 ^{b}	52.49 ± 0.75 ^{b}	57.85 ± 1.92 ^{ab}	55.70 ± 0.35 ^{a}	57.01 ± 0.80 ^{a}
48 h	44.41 ± 0.76 ^{c}	46.42 ± 0.46 ^{c}	53.07 ± 0.76 ^{abcd}	50.49 ± 1.04 ^{b}	51.73 ± 1.24 ^{b}
72 h	29.00 ± 0.31 ^{d}	38.77 ± 0.41 ^{d}	48.85 ± 0.88 ^{d}	43.84 ± 0.19 ^{c}	47.98 ± 0.68 ^{cd}
96 h	10.39 ± 0.36 ^{e}	28.82 ± 0.94 ^{e}	50.52 ± 0.64 ^{bcd}	39.74 ± 0.57 ^{d}	48.78 ± 0.97 ^{cd}
120 h	1.99 ± 0.06 ^{f}	16.79 ± 1.75 ^{f}	47.47 ± 0.62 ^{d}	33.87 ± 1.02 ^{e}	46.31 ± 0.53 ^{d}
144 h	1.97 ± 0.05 ^{g}	4.02 ± 1.17 ^{g}	48.17 ± 0.56 ^{d}	28.00 ± 0.51 ^{f}	46.47 ± 0.45 ^{d}
168 h	1.99 ± 0.12 ^{g}	1.77 ± 0.03 ^{g}	48.51 ± 0.50 ^{d}	21.55 ± 0.75 ^{g}	47.61 ± 0.27 ^{cd}
192 h	2.01 ± 0.00 ^{g}	1.85 ± 0.07 ^{g}	52.06 ± 1.29 ^{abcd}	13.71 ± 0.92 ^{h}	48.29 ± 0.44 ^{cd}
216 h	1.92 ± 0.02 ^{g}	1.80 ± 0.07 ^{g}	49.80 ± 0.93 ^{cd}	3.44 ± 0.68 ^{i}	48.96 ± 0.77 ^{bcd}
240 h	2.04 ± 0.13 ^{g}	1.89 ± 0.13 ^{g}	57.13 ± 6.27 ^{abc}	2.77 ± 0.12 ^{i}	49.60 ± 1.26 ^{bc}
**Squalene content (mg/g)**					
24 h	1.73 ± 0.59 ^{bc}	2.93 ± 0.07 ^{e}		2.85 ± 0.15 ^{d}	
48 h	9.90 ± 1.65 ^{a}	3.60 ± 0.68 ^{de}		6.27 ± 0.78 ^{bc}	
72 h	8.55 ± 1.74 ^{a}	5.11 ± 0.60 ^{cd}		7.58 ± 0.57 ^{abc}	
96 h	4.86 ± 1.01 ^{b}	6.35 ± 0.33 ^{c}		8.12 ± 0.33 ^{ab}	
120 h	2.27 ± 0.56 ^{bc}	8.63 ± 0.58 ^{b}		8.68 ± 1.05 ^{a}	
144 h	1.94 ± 0.37 ^{bc}	11.07 ± 0.52 ^{a}		8.20 ± 0.30 ^{ab}	
168 h	1.49 ± 0.39 ^{c}	11.95 ± 0.64 ^{a}		8.20 ± 0.37 ^{ab}	
192 h	1.18 ± 0.30 ^{c}	11.35 ± 0.81 ^{a}		7.81 ± 0.70 ^{abc}	
216 h	0.91 ± 0.30 ^{c}	10.29 ± 0.69 ^{a}		7.95 ± 0.69 ^{abc}	
240 h	0.89 ± 0.14 ^{c}	10.42 ± 0.39 ^{a}		5.96 ± 0.29 ^{c}	
**Squalene yield (mg/L)**					
24 h	6.08 ± 2.18	11.45 ± 0.49 ^{f}		7.22 ± 0.61 ^{f}	
48 h	76.28 ± 14.23	26.57 ± 4.52 ^{f}		23.85 ± 2.91 ^{ef}	
72 h	111.29 ± 19.96	47.72 ± 4.45 ^{ef}		48.64 ± 2.25 ^{de}	
96 h	88.94 ± 18.26	76.47 ± 3.93 ^{e}		62.33 ± 11.5 ^{cd}	
120 h	46.69 ± 11.91	116.26 ± 10.92 ^{d}		75.76 ± 8.42 ^{bcd}	

	M50	M50 + N	M50 - N	M50 – N +E	E
144 h	39.98 ± 7.54	187.20 ± 15.31 [c]		82.86 ± 11.84 [bc]	
168 h	30.42 ± 8.27	227.40 ± 11.67 [a]		78.46 ± 2.70 [bc]	
192 h	23.75 ± 6.58	200.06 ± 17.67 [abc]		97.07 ± 9.68 [ab]	
216 h	20.46 ± 6.68	173.26 ± 12.89 [bc]		123.79 ± 14.11 [a]	
240 h	17.67 ± 2.73	177.38 ± 10.59 [bc]		89.14 ± 3.14 [bc]	
**pH**					
0 h	6.68 ± 0.14 [b]	6.85 ± 0.00 [de]	7.35 ± 0.01 [ab]	6.84 ± 0.02 [b]	6.72 ± 0.02 [f]
24 h	6.73 ± 0.02 [b]	6.95 ± 0.01 [cde]	7.14 ± 0.01 [ab]	6.58 ± 0.04 [c]	6.48 ± 0.00 [e]
48 h	6.81 ± 0.00 [b]	6.81 ± 0.04 [de]	6.99 ± 0.37 [b]	6.24 ± 0.07 [d]	6.90 ± 0.04 [d]
72 h	6.87 ± 0.00 [b]	6.78 ± 0.00 [e]	7.37 ± 0.07 [ab]	6.16 ± 0.01 [d]	7.33 ± 0.03 [bc]
96 h	6.88 ± 0.01 [b]	6.82 ± 0.02 [de]	7.43 ± 0.01 [a]	6.12 ± 0.00 [d]	7.56 ± 0.01 [a]
120 h	7.37 ± 0.02 [a]	6.99 ± 0.03 [cd]	7.47 ± 0.01 [a]	6.09 ± 0.01 [d]	7.47 ± 0.03 [a]
144 h	7.41 ± 0.01 [a]	7.12 ± 0.00 [c]	7.52 ± 0.01 [a]	6.12 ± 0.02 [d]	7.33 ± 0.03 [b]
168 h	7.43 ± 0.03 [a]	7.53 ± 0.06 [b]	7.49 ± 0.03 [a]	6.25 ± 0.00 [d]	7.23 ± 0.02 [bc]
192 h	7.51 ± 0.08 [a]	7.89 ± 0.11 [a]	7.52 ± 0.02 [a]	6.47 ± 0.04 [c]	7.22 ± 0.06 [c]
216 h	7.56 ± 0.07 [a]	8.06 ± 0.03 [a]	7.47 ± 0.04 [a]	6.85 ± 0.11 [b]	7.32 ± 0.01 [bc]
240 h	7.55 ± 0.07 [a]	7.95 ± 0.12 [a]	7.52 ± 0.01 [a]	7.35 ± 0.14 [a]	7.31 ± 0.02 [c]
